# Optimization two-qubit quantum gate by two optical control methods in molecular pendular states

**DOI:** 10.1038/s41598-022-18967-2

**Published:** 2022-09-01

**Authors:** Jin-Fang Li, Jie-Ru Hu, Feng Wan, Dong-Shan He

**Affiliations:** 1 Department of Physics and Electronic Engineering, Xianyang Normal University, Shaanxi, 712000 China; 2 State Key Laboratory of Precision Spectroscopy, Department of Physics, East China Normal University, Shanghai, 200062 China

**Keywords:** Quantum simulation, Qubits

## Abstract

Implementation of quantum gates are important for quantum computations in physical system made of polar molecules. We investigate the feasibility of implementing gates based on pendular states of the molecular system by two different quantum optical control methods. Firstly, the Multi-Target optimal control theory and the Multi-Constraint optimal control theory are described for optimizing control fields and accomplish the optimization of quantum gates. Numerical results show that the controlled NOT gate (CNOT) can be realized under the control of above methods with high fidelities (0.975 and 0.999) respectively. In addition, in order to examine the dependence of the fidelity on energy difference in the same molecular system, the SWAP gate in the molecular system is also optimized with high fidelity (0.999) by the Multi-Constraint optimal control theory with the zero-area and constant-fluence constraints.

## Introduction

Control and implementation of quantum logical gates are important for quantum computing^1–3^. In 2002, DeMille first proposed a model to do quantum calculation based on molecular system and pointed that quantum bits can be seen as the direction of dipole moment up and down along the static field in ultra-cold polar diatomic molecular system^4^. Meanwhile, the qubits can be combined with the dipole-dipole interaction so that the quantum logical gates can be generated reasonably in decoherence time^4^. Since then, the proposals have attracted more and more attention about performing quantum gates in molecular system^5–22^. The diverse structures of polar molecule are chosen as the quantum basis to optimize quantum logical gates under the control of laser pulse. For example, the authors have did much quantum calculation based on molecular vibrational states^5–10^. In vibrational states of C$_{2}$H$_{2}$ system, two different IR-active model can be seen as quantum basis, so that the NOT and CNOT can be achieved with the fidelity more than 0.90 under the control of external field^5^, while the fidelity is 0.96 for generating Hadamard gate^6^. Based on the rotational and vibrational states, the authors have discussed^11–14^ in NH$_{3}$ vibrational states with high fidelity 0.93 to achieve CNOT^11^, and in electronic ground states of Na$_{2}$ and Li$_{2}$ with 0.83 fidelity to achieve DJ algorithm^12^; following, with two dipolar system NaBr-NaBr, based on the rotational and electronic ground states, the value of fidelity can reach 0.979. Recently, the authors demonstrate the high fidelities of entanglement between the rotational states of a molecular ion and the internal states of a atomic ion^15^. In Ref.^16^, CNOT gate is generated based on quantum Zeno dynamics in order to make the system evolve into the target state between two atoms. And the authors studied the quantum adder and subtractor with fidelity 0.97 by coding on the molecular vibrational states^23–26^. Even in hyperfine levels of electronic ground states, the quantum computing model based on the electronic ground states of H$_{2}$, LiH and H$_{2}$O also have been studied^27^.

Furthermore, the pendular state is formed based on rotational states when the molecules are trapped and partially oriented by external electric fields^28,29^ and the special state can afford more information of polar molecule about alignment and orientation^30,31^. Much attention have been attracted on the quantum entanglement and quantum computing in pendular systems^28,32,33^. Herschbach et al has calculated the quantum concurrence in pendular state formed by two dipoles, in addition, when the dipole moment and rotational constant are set as variable, it can be demonstrated that the quantum logical gates can be operated by calculating the concurrence between two pendular states^33^. Specifically, in the pendular system of SrO-SrO system, the single-qubit gate (NOT) and two-qubit logical gate (CNOT) can be achieved with the high fidelity of the values 0.985 and 0.975 respectively^28^. In general, a two-level system is basic and simple system to perform the quantum computing when the qubits $|0\rangle$ and $|1\rangle$ are considered as quantum basis for single-qubit gate^34–37^. By same principle, a four-level system can be implemented for two-qubit gate if the quantum basis include $|00\rangle$, $|01\rangle$, $|10\rangle$ and $|11\rangle$ which are complete and orthogonal^28^. Both of the two-level system and four-level system can optimize the quantum gates due to the energy difference between different energy levels in molecular systems.

Quantum optical control methods provide a powerful tool to make the quantum initial states evolve to the final states with control fields^38–42^. Rabitz et al proposed Multi-Target Optital Control Theory (MT-OCT) based on the coupled Schrödinger equations^43,44^, and the theory aims to solve the two-point boundary equations step by step with many iterations. This method has attracted attention extensively for the specific physical molecular system^5,6,8,10,12,13,23–25,28^. Later, Rabitz et al have proposed the other quantum optimal control theory based on optimization of unitary operation^45^. The core of this theory is optimization the operation which satisfied Schrödinger equation, and the control field updates with the increasing of iteration. Finally, the unitary operation is applied in any initial state to optimize quantum logical gate to the final state^45–49^. To satisfy conditions named zero-area and constant-fluence of control pulse, Shu and collaborators promoted this theory by adding constraints on control pulse^46–48^ which is named as Multiple Constraint Quantum Optical Control (MC-OCT). With two important constraints on the optimized control fields during optimization, i.e., no-dc component and constant pulse fluence, then the time-dependent control protocol is keeping the pulse energy unchanged as compared with the initial inputs.

Based on the above analysis, in our work, we apply the above two quantum optical control methods to guide the control fields for optimization of two-qubit quantum logical gates. Particularly, we aim to get the CNOT and SWAP gates and try to find the priority method for optimization of two-qubit gate. The results show that both of the two quantum optical control methods can achieve the high efficiency to get the optimal control fields in the given pendular system. Compared to the results in Ref.^28^, the higher fidelity 0.999 for CNOT and SWAP quantum gates can be reached by MC-OCT in our work, so that an admissible error lower than $10^{-3}$ can be achieved in molecular system theoretically. Furthermore, the simulation difference of optimization situations is studied as well as the energy difference between different quantum basis in pendular system.

## Results

### Pendular system consisted of polar molecules

We consider two polar diatomic molecules trapped in an external static electric field $(\epsilon )$, due to the Stark interaction, the system can be seen as pendular states resulting from mixing of the field-free rotational states^28^. In rigid-rotor approximation, the Hamiltonian of the two trapped molecules can be written as following

1 $$\begin{aligned} \hat{H}_{1}=B \hat{J}^{2}-\mu \epsilon _{1 }\cos \theta _{1} \end{aligned}$$

and

2 $$\begin{aligned} \hat{H}_{2}=B \hat{J}^{2}-\mu \epsilon _{2 }\cos \theta _{2}, \end{aligned}$$

where *B* is the rotational constant, $\mu$ is the permanent dipole moment and $\hat{J}$ is the angular momentum operator. $\theta _{1}$ and $\theta _{2}$ represent the polar angles between the dipole moment and the static field direction. The first molecule experiences an external static field of $\epsilon _{1}$ whereas the second molecule experiences an external static field of $\epsilon _{2}$. The external static field contains a gradient in position of the two molecules allowing spectroscopic addressing of each site^4^. The eigenstates of $\hat{H}_{1}$ and $\hat{H}_{2}$ are designated as pendular states resulting from the mixing of the field-free rotational states by the Stark effect.

As proposed in Ref.^4^, basic qubits are chosen as two molecular states. We can choose the ground state and second excited state $\vert \tilde{J}_{0}\rangle$ and $\vert \tilde{J}_{2}\rangle$ as the two pendular states, which can be described as superpositions of spherical harmonics^28^

3 $$\begin{aligned} \langle \theta ,\varphi \vert \tilde{J}_{0} \rangle =\sum \limits _{j} a_{j} Y_{j,0}(\theta ,\varphi ); \langle \theta ,\varphi \vert \tilde{J}_{1} \rangle =\sum \limits _{j} c_{j} Y_{j,0}(\theta ,\varphi ). \end{aligned}$$

$a_{j}$ and $c_{j}$ are the coefficients of spherical harmonics and $\varphi =0$. In the absence of a control field, the total Hamiltonian of the two trapped molecules in the basis of the qubit pendular states can be written as a $4\times 4$ matrix

4 $$\begin{aligned} \hat{H}_{0}=\hat{H}_{1} \otimes \hat{I}+ \hat{I} \otimes \hat{H}_{2}+ \hat{V}_{dd}, \end{aligned}$$

where $\hat{I}$ is a 2$\times$2 identity matrix. The eigenenergies $E^{0}_{1}$ and $E^{1}_{1}$ of the Hamiltonian $\hat{H}_{1}$ are calculated based on the quantum basis in Eq. (3), and $E^{0}_{2}$ and $E^{1}_{2}$ of $\hat{H}_{2}$ for the second molecule are calculated in the same way. The specific expressions are given as

5 $$\begin{aligned} \hat{H}_{1}=\left( \begin{array}{cc} E^{0}_{1} &{} 0 \\ 0 &{} E^{1}_{1} \end{array} \right) ; \hat{H}_{2}=\left( \begin{array}{cc} E^{0}_{2} &{} 0 \\ 0 &{} E^{1}_{2} \end{array} \right) . \end{aligned}$$

The last term $\hat{V}_{dd}$ in Eq. (4) describes the dipole-dipole interaction given by^50^

6 $$\begin{aligned} \hat{V}_{dd}= \dfrac{\mu ^{2}}{2\pi \varepsilon _{0}r_{12}^{3}}(1-3 \cos ^{2}\alpha ) \cdot \cos \theta _{1}\cdot \cos \theta _{2}, \end{aligned}$$

where, $\mu$ is permanent dipole moment, ${r_{12}}$ is the distance between the two molecules, and $\alpha$ is the angle between the array axis $\hat{r}_{12}$ and the static electric field direction. Specifically, here for convenience calculation, $\alpha$ is set as 90 degree, then the matrix expression of $\hat{V}_{dd}$ is given as

7 $$\begin{aligned} \hat{V}_{dd}= \dfrac{\mu ^{2}}{2\pi \varepsilon _{0}r_{12}^{3}} \left[ \left( \begin{array}{cc} C^{1}_{0} &{} C^{1}_{x} \\ C^{1*}_{x} &{} C^{1}_{1} \end{array} \right) \otimes \left( \begin{array}{cc} C^{2}_{0} &{} C^{2}_{x} \\ C^{2*}_{x} &{} C^{2}_{1} \end{array} \right) \right] , \end{aligned}$$

where the elements are calculated as $C^{1}_{0} =\left\langle \tilde{J}_{0} \right| \cos \theta _{1} \left| \tilde{J}_{0} \right\rangle , C^{1}_{1} =\left\langle \tilde{J}_{1} \right| \cos \theta _{1}\left| \tilde{J}_{1} \right\rangle , C^{1}_{x} =\left\langle \tilde{J}_{0} \right| \cos \theta _{1} \left| \tilde{J}_{1} \right\rangle , C^{1*}_{x} =\left\langle \tilde{J}_{1} \right| \cos \theta _{1}\left| \tilde{J}_{0} \right\rangle , C^{2}_{0} =\left\langle \tilde{J}_{0} \right| \cos \theta _{2} \left| \tilde{J}_{0} \right\rangle , C^{2}_{1} =\left\langle \tilde{J}_{1} \right| \cos \theta _{2}\left| \tilde{J}_{1} \right\rangle , C^{2}_{x} =\left\langle \tilde{J}_{0} \right| \cos \theta _{2}\left| \tilde{J}_{1} \right\rangle ,$ and $C^{2*}_{x} =\left\langle \tilde{J}_{1} \right| \cos \theta _{2} \left| \tilde{J}_{0} \right\rangle .$

So the specific elements of Hamiltonian in Eq. (4) can be written as

8 $$\begin{aligned} H_{0}= \left( \begin{array}{cccc} E^{0}_{1}+E^{0}_{2}+C^{1}_{0}C^{2}_{0} &{} C^{1}_{0}C^{2}_{x} &{} C^{1}_{x}C^{2}_{0} &{} C^{1}_{x}C^{2}_{x} \\ C^{1}_{0}C^{2*}_{x} &{} E^{0}_{1}+E^{1}_{2}+C^{1}_{0}C^{2}_{1} &{} C^{1}_{x}C^{2*}_{x} &{} C^{1}_{x}C^{2}_{1} \\ C^{1*}_{x}C^{2}_{0} &{} C^{1*}_{x}C^{2}_{x} &{} E^{1}_{1}+E^{0}_{2}+C^{1}_{1}C^{2}_{0} &{} C^{1}_{1}C^{2}_{x} \\ C^{1*}_{x}C^{2*}_{x} &{} C^{1*}_{x}C^{2}_{1} &{} C^{1}_{1}C^{2*}_{x} &{} E^{1}_{1}+E^{1}_{2}+C^{1}_{1}C^{2}_{1} \end{array} \right) . \end{aligned}$$

### Optimization CNOT by multi-target optical control theory

In this section, we will optimize the specific quantum gate by applying the method named Multi-Target Optical Control Theory (MT-OCT). In Ref.^28^, $\lbrace \vert 00\rangle$ $\vert 01\rangle$  $\vert 10\rangle$ $\vert 11\rangle \rbrace$ are a set of orthogonal and complete quantum basis vectors corresponding to pendular system. In the following simulation, we choose the same quantum basis as the four lowest energy levels of molecular pendular system. The specific form can be written as $\lbrace \vert \tilde{J}_{0}\rangle \otimes \vert \tilde{J}_{0}\rangle \rightarrow \vert 00 \rangle , \vert \tilde{J}_{0}\rangle \otimes \vert \tilde{J}_{1}\rangle \rightarrow \vert 01 \rangle , \vert \tilde{J}_{1}\rangle \otimes \vert \tilde{J}_{0}\rangle \rightarrow \vert 1 0\rangle , \vert \tilde{J}_{1}\rangle \otimes \vert \tilde{J}_{1}\rangle \rightarrow \vert 1 1\rangle \rbrace$ in the SrO-SrO pendular system, Combined the Eq. (8) with the control pulse, then the expression of time-dependent Hamiltonian can be expressed as

9 $$\begin{aligned} \hat{H}(t)=\hat{H}_{0}- \mu \hat{E}(t), \end{aligned}$$

The inherent dipole moment is $\mu =8.9$ Debye (3.5 a.u.), $\hat{H}_{0}$ is the Hamiltonian of Eq. (4) and the rotational constant is $B=0.33 cm^{-1}$ ($1.5 \times 10^{-6}$ a.u.). The static electric field is set as $\epsilon _{1}=4.4$ kV/cm ($0.86 \times 10^{-6}$ a.u.) and $\epsilon _{2}=6.6$ kV/cm ($1.28 \times 10^{-6}$ a.u.) in Eqs. (1) and (2). Then the address of two molecules will have gradient difference. Here, it should be noticed that the $\mu \epsilon /B$ is unitless. According to the Ref.^4^, the value of $\mu \epsilon /B$ changes between 2 and 5 reasonably, so we choose the values as $\mu \epsilon _{1}/B=2$ and $\mu \epsilon _{2}/B=3$ during the following simualtion. In addition, to facilitate the optimization process, the distance between two molecule are set as $r_{12}=50 nm$ ($9.45\times 10^{2}$ a.u.). The initial pulse is set as $E(t)=S(t)E_{0} \cos (2 \pi \upsilon t)$, where *S*(*t*) is envelope function. The general form is $S(t)=\sin ^{2}(\pi t/T)$, $E_{0}$ is the amplitude, and the initial value is set as 1.5 kV/cm (2.9$\times 10^{-7}$ a.u.) and the pulse duration T is set as 65 ns (2.6$\times 10^{9}$ a.u.). Based on the above given data, we take the fixed step for Ruge–Kutta method and the Lagrange compensation factor is $\alpha _{0}$=5$\times 10^{6}$ (unitless) and the time step is given as $\Delta$t=0.25 ps. In addition, the cosine matrix elements are $C^{1}_{0}=0.4677$, $C^{1}_{1}=0.4250$, $C^{1}_{x}=0.3599$, $C^{2}_{0}=0.5726$, $C^{2}_{1}=0.5913$, $C^{2}_{x}=0.4867$, and the specific calculation method can be found in Ref.^33^.

The two-qubit quantum gate CNOT and SWAP have the specific form as following

10 $$\begin{aligned} CNOT= & {} \left[ \begin{array}{cccc} 1 &{} 0 &{} 0 &{} 0 \\ 0 &{} 1 &{} 0 &{} 0 \\ 0 &{} 0 &{} 0 &{} 1 \\ 0 &{} 0 &{} 1 &{} 0 \\ \end{array} \right] \end{aligned}$$

11 $$\begin{aligned} SWAP= & {} \left[ \begin{array}{cccc} 1 &{} 0 &{} 0 &{} 0 \\ 0 &{} 0 &{} 1 &{} 0 \\ 0 &{} 1 &{} 0 &{} 0 \\ 0 &{} 0 &{} 0 &{} 1 \\ \end{array} \right] \end{aligned}$$

According to the principle of CNOT operation, when CNOT is applied on the four quantum basis, the population of $\vert 00 \rangle$ and $\vert 01 \rangle$ will keep the original distributions. However, the population of $\vert 10 \rangle$ and $\vert 11 \rangle$ will exchange distributions for each other. Here, in order to make the simulation more efficiently, we choose an initial state to optimize the CNOT based on the above pendular system. For example, when the initial state is $[\sin (\pi /3)\vert 00 \rangle +\cos (\pi /3)\vert 10 \rangle ]e^{-i\phi }$, after the operation of CNOT, then the final state should be $[\sin (\pi /3)\vert 00 \rangle +\cos (\pi /3)\vert 11 \rangle ]e^{-i\phi }$ in theory. Whereas the initial population should be zero for $\vert 01 \rangle$ and $\vert 11 \rangle$.

**Figure 1:**
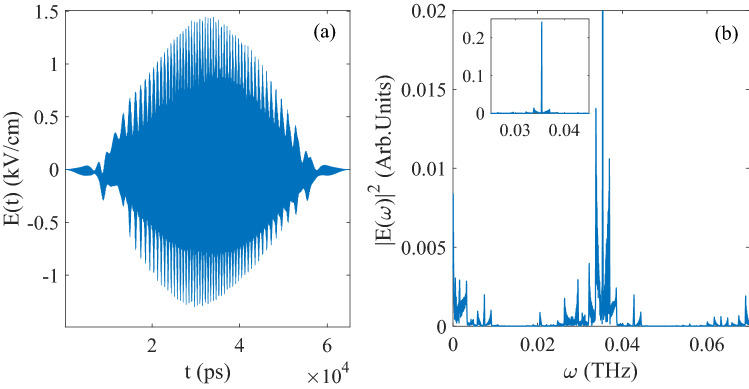
(**a**) The evolution of control pulse as function of time corresponding to CNOT gate; (**b**) The Fourier transform of the control pulse in (**a**).

In Fig.1a, the optimized pulse of this initial state operated by CNOT is given as function of evolution time and the amplitude of the pulse is around 1.5kV/cm. From Fig.1b, it can be seen the result of central frequency is 0.035 THz ($1.1785 cm^{-1}$), and the central frequency is the pendular energy level difference which is corresponding to the quantum basis $\vert 10 \rangle$ and $\vert 11 \rangle$ and satisfies the condition of resonance (the energy value is difference between $1.5726 cm^{-1}$ and $0.3939 cm^{-1}$).

**Figure 2:**
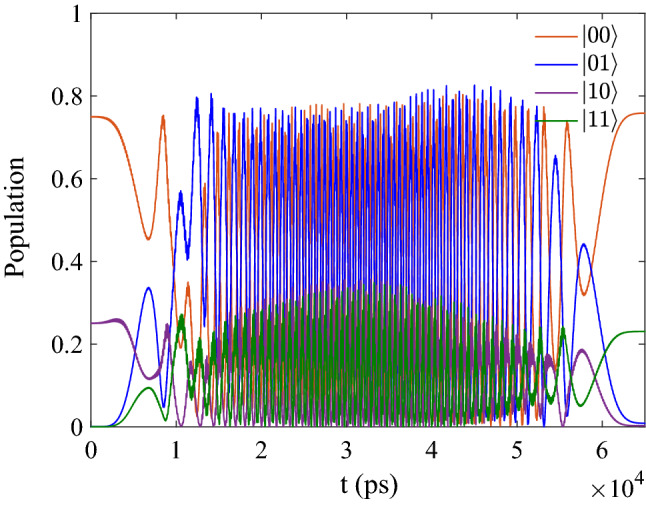
The evolution of population as function of time corresponding to the initial state $[\sin (\pi /3)\vert 00 \rangle +\cos (\pi /3)\vert 10 \rangle ]e^{-i\phi }$.

In Fig. 2, we give the initial state which has the form $[\sin (\pi /3)\vert 00 \rangle +\cos (\pi /3)\vert 10 \rangle ]e^{-i\phi }$ as the function of time when the initial phase is ${\phi }=60$ deg. And the final population should be 0.75 for $\vert 00 \rangle$, 0.00 for $\vert 01 \rangle$, 0.00 for $\vert 10 \rangle$ and 0.25 for $\vert 11 \rangle$ in theory. When we optimize the CNOT in this molecular system by MT-OCT, the fidelity F can reach 0.975 based on the above parameters, then the final population is 0.7582 $\vert 00 \rangle$, 0.0086 $\vert 01 \rangle$, 0.0026 $\vert 10 \rangle$ and 0.2304 $\vert 11 \rangle$, thus the CNOT is operated correctly.

**Figure 3:**
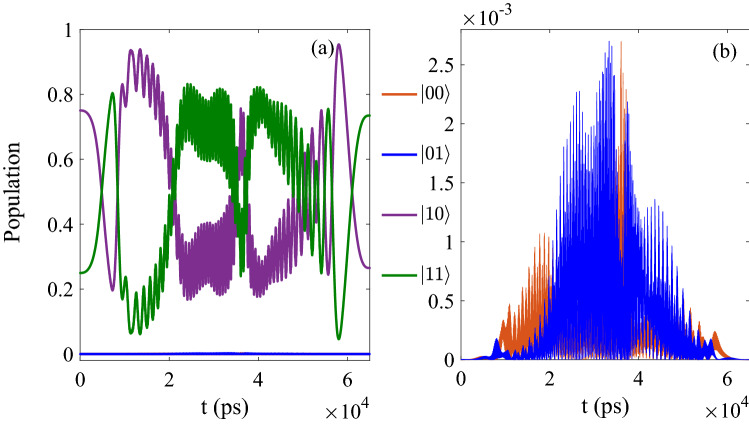
(**a**) The evolution of population as function of time corresponding to the initial state $[\sin (\pi /3)\vert 10 \rangle +\cos (\pi /3)\vert 11 \rangle ]e^{-i\phi }$; (**b**) The specific evolution of the basis $[\vert 10 \rangle$ and $\vert 11 \rangle ]e^{-i\phi }$.

In Fig. 3, we give the initial state which has the form $[\sin (\pi /3)\vert 10 \rangle +\cos (\pi /3)\vert 11 \rangle ]e^{-i\phi }$ as the function of time. And the final population should be 0.00 for $\vert 00 \rangle$, 0.00 for $\vert 01 \rangle$, 0.25 for $\vert 10 \rangle$ and 0.75 for $\vert 11 \rangle$ in theory. After the optimization, the final population is 0.0073 for $\vert 00 \rangle$, 0.0034 for $\vert 01 \rangle$, 0.2641 for $\vert 10 \rangle$ and 0.7251 for $\vert 11 \rangle$, the result is closed to the theoretical expected value.

Compared the simulations to the theoretical values, it can be seen the results are very closed to each other which means the optimization of CNOT is successful. Meanwhile, the fidelity *F* in Eq. (16) is 0.975 while the ideal value should be 1. In order to improve the fidelity (objective) and precision of optimization, we will optimize CNOT through MC-OCT thereinafter.

### Optimization CNOT by multi-constraint optical control theory

In this section, we will apply the other method named Multi-Constraint Optical Control Theory (MC-OCT) to do the optimization. And the two-qubit CNOT gate will be optimized based on the the pendular state of SrO-SrO system. The initial control field is set as $E(t)=S(t)E_{0} \cos ^{2}(2 \pi \upsilon t)$, where *S*(*t*) is the envelope function, and the specific form is $S(t)=\sin (\pi t /T)^{2}$. Where *T* is the total time and $T=$ 65 ns ($2.60\times 10^{9}$ a.u.). During the optimization, $\Delta t=0.06$ ps ($2.4\times 10^{3}$ a.u.), the iteration step is $\Delta s=1.5\times 10^{-7}$. The initial value of dummy variable *s* is zero, $\Delta s$ can be modulated to find the most optimal control fields *E*(*s*, *t*). $E_{0}$ and $\upsilon$ represent amplitude and frequency of control pulse. $E_{0}=5\times 10^{-2}$ kV/cm ($1\times 10^{-8}$ a.u.).

**Table 1:** The optimization results of CNOT.

	Initial state	Final state	F
(i)	$1/2(\vert 00 \rangle +\vert 01 \rangle +$	$1/2(\vert 00 \rangle e^{-i\phi _{1}} +\vert 01 \rangle e^{-i\phi _{2}}+$	0.999
	$\vert 10 \rangle +\vert 11 \rangle )e^{-i\phi }$	$\vert 11 \rangle e^{-i\phi _{3}}+\vert 10 \rangle e^{-i\phi _{4}})$	
(ii)	$[\sin (\pi /3)\vert 00 \rangle +$	$\sin (\pi /3)\vert 00 \rangle e^{-i\phi _{1}}+$	0.999
	$\cos (\pi /3)\vert 10 \rangle ]e^{-i\phi }$	$\cos (\pi /3)\vert 11 \rangle e^{-i\phi _{2}}$	
(iii)	$[\sin (\pi /3)\vert 10 \rangle +$	$\sin (\pi /3)\vert 11 \rangle e^{-i\phi _{1}}+$	0.999
	$\cos (\pi /3)\vert 11 \rangle ]e^{-i\phi }$	$\cos (\pi /3)\vert 10 \rangle e^{-i\phi _{2}}$	

**Figure 4:**
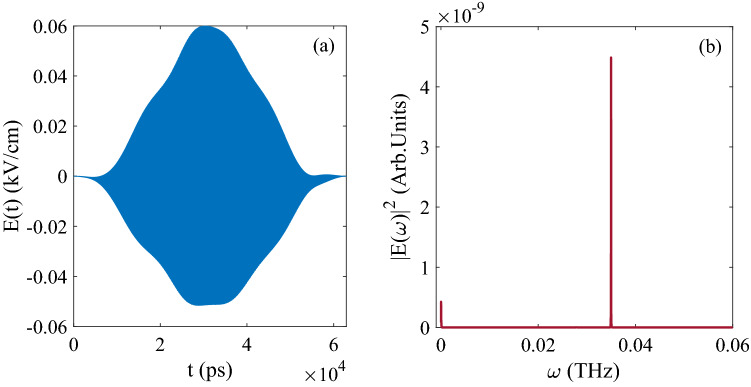
(**a**) The evolution of control pulse as function of time; (**b**) The Fourier transform of the pulse in (**a**).

In Table 1, we give the results of different initial states operated by CNOT. F is abbreviation of $F(U_{T})$ in Eq. (18), the results is 0.999 which approaches to the ideal value 1. During the calculation and simulation in this section, the initial phase $\phi$ is set as 60 (deg). The optimized pulse can be plotted as the function of time in Fig. 4a, and the amplitude of pulse is around 0.06 kV/cm. Besides, it can be seen that the central frequency is 0.035 THz from Fig. 4b, and that means the pulse satisfies the condition of resonance and have the same performance under the control of MT-OCT.

**Figure 5:**
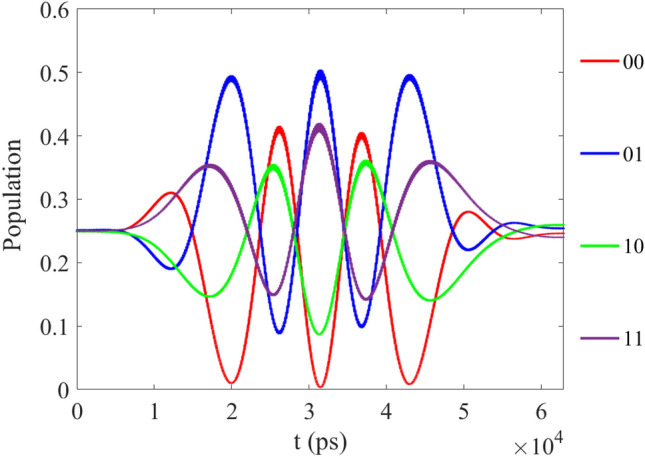
The evolution of population as function of time corresponding to the initial state 0.25 $\vert 00 \rangle$ + 0.25 $\vert 01 \rangle$+0.25 $\vert 10 \rangle$+0.25 $\vert 11 \rangle$.

In Fig. 5, when the initial state is given as $1/2[\vert 00 \rangle +\vert 10 \rangle +\vert 10 \rangle +\vert 11 \rangle ]e^{-i\phi }$, after the operation of CNOT, the final population is 0.2485 for $\vert 00 \rangle$, 0.2514 for $\vert 01 \rangle$, 0.2313 for $\vert 10 \rangle$ and 0.2688 for $\vert 11 \rangle$, and the ideal value should be 0.25 for $\vert 00 \rangle$, 0.25 for $\vert 01 \rangle$, 0.25 for $\vert 10 \rangle$ and 0.25 for $\vert 11 \rangle$) in theory. And the fidelity can reach 0.999 while the ideal value is 1.

**Figure 6:**
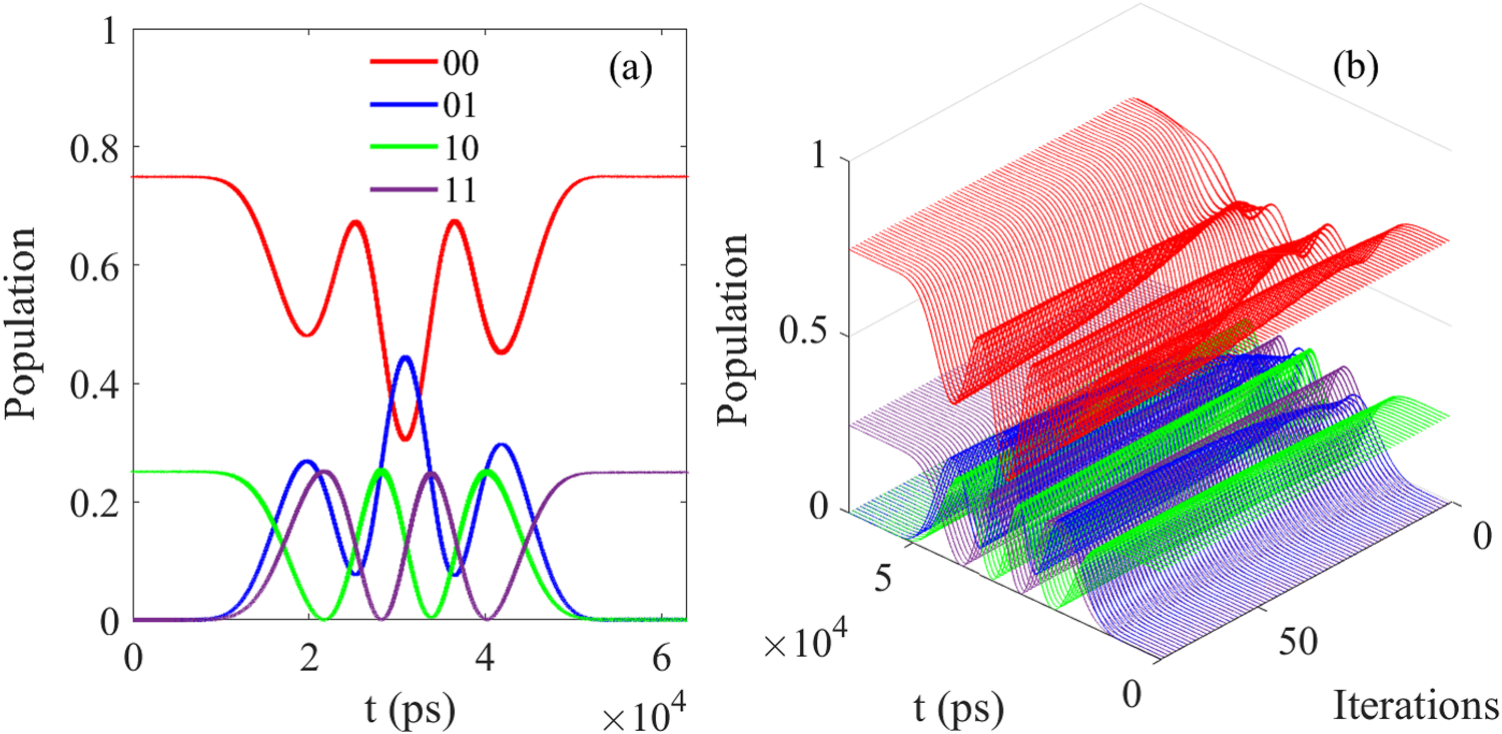
The results corresponding to 1 (ii); (**a**) The evolution of control pulse as function of time; (**b**) The evolution of control pulse as function of time and iterations.

Then we check the other initial state to optimize the CNOT which is similar to the initial state above by the MT-OCT, and in Fig. 6, when the initial state is $[\sin (\pi /3)\vert 00 \rangle + \cos (\pi /3)\vert 10 \rangle ]e^{-i\phi }$, after the operation of CNOT, the final population is 0.7482 for $\vert 00 \rangle$, 0.0017 for $\vert 01 \rangle$, 0.0004 for $\vert 10 \rangle$ and 0.2498 for $\vert 11 \rangle$, which is closed to the ideal value 0.75 for $\vert 00 \rangle$, 0.00 for $\vert 01 \rangle$, 0.00 for $\vert 10 \rangle$ and 0.25 for $\vert 11 \rangle$ in theory while the fidelity is also 0.999.

**Figure 7:**
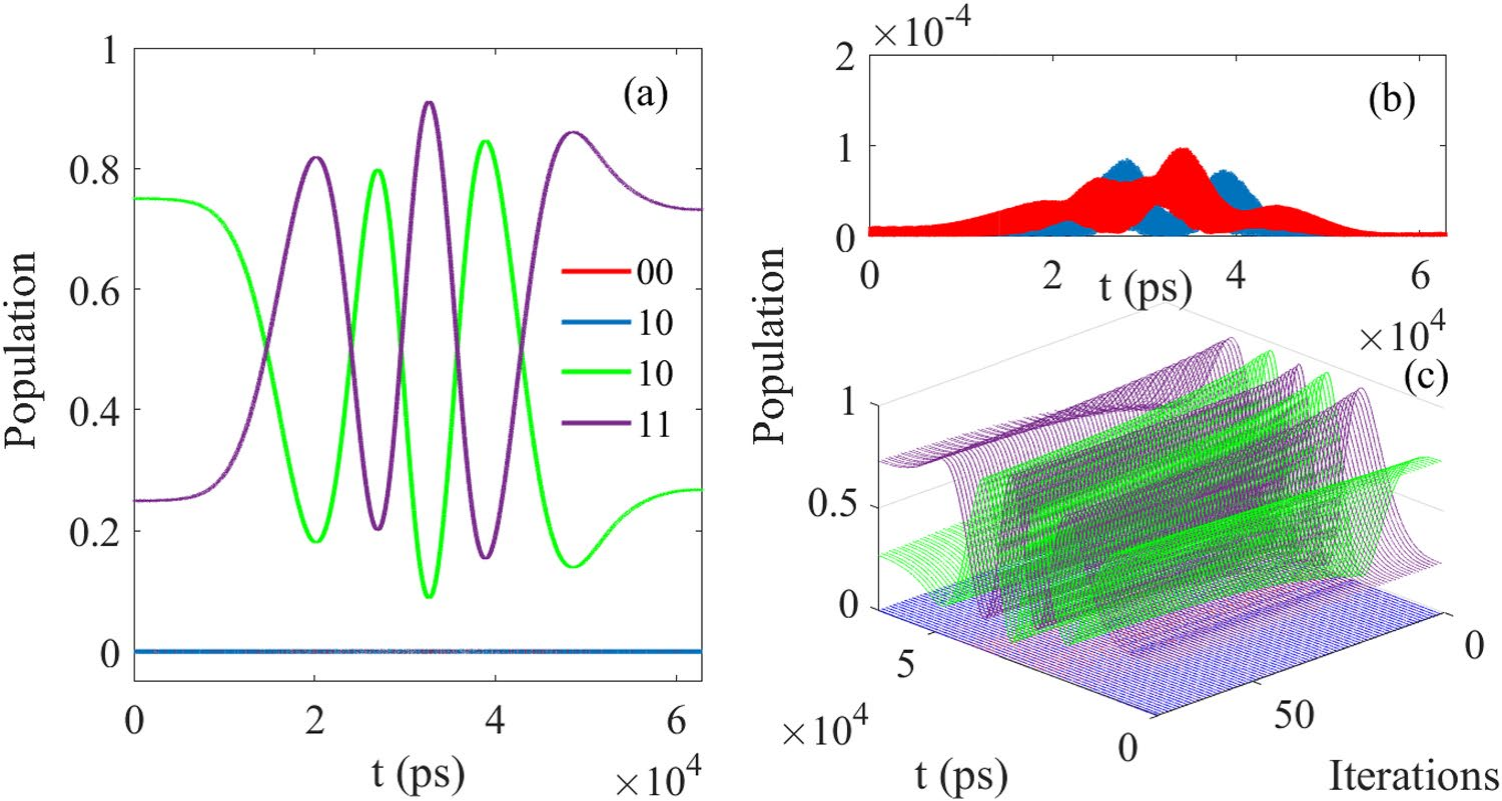
The results corresponding to Table 1(iii); (**a**) The evolution of control pulse as function of time; (**b**) The evolution of control pulse as function of time $\vert 00 \rangle$ and $\vert 01 \rangle ]e^{-i\phi }$; (**c**) The evolution of control pulse as function of time and iterations.

And in Fig. 7, when the initial state is $[\sin (\pi /3)\vert 10 \rangle + \cos (\pi /3)\vert 11 \rangle ]e^{-i\phi }$, after the operation of CNOT, the final population is 0.0000 for $\vert 00 \rangle$, 0.0000 for $\vert 01 \rangle$, 0.2581 for $\vert 10 \rangle$ and 0.7476 for $\vert 11 \rangle$, and the ideal value should be 0.0000 $\vert 00 \rangle$, 0.00001 $\vert 01 \rangle$, 0.2581 $\vert 10 \rangle$ and 0.7476 $\vert 11 \rangle$ in theory. When the result is compared to the values of 0.0073 for $\vert 00 \rangle$, 0.0034 for $\vert 01 \rangle$, 0.2641 for $\vert 10 \rangle$ and 0.7251 for $\vert 11 \rangle$, it can be seen that the former is closer to the theoretical value.

Based on this pendular system, we optimize the CNOT by two different methods named MT-OCT and MC-OCT respectively. Compared the results of both processing, for the same initial state, the fidelity is different and the values are 0.975 in MT-OCT and 0.999 in MC-OCT respectively. Obviously, the more higher fidelity is maintained in MC-OCT. Meanwhile, the iterations for the running time of Code are needed longer in the MT-OCT than in the MC-OCT.

### Optimization SWAP by multi-constraint optical control theory

Based on the ideal optimization of CNOT, we apply the MC-OCT to optimize the two-qubit logical gate named SWAP. The initial pulse is set as $E(t)=S(t)E_{0} \cos ^{2}(2 \pi \upsilon t)$, $S(t)=\sin (\pi t /T)^{2}$. The total time is *T*=$1.3\times 10^{3}$ ns. During the optimization, $\Delta t=0.06$ ps ($2.4\times 10^{3}$ a.u.), the iteration step is $\Delta s=1.5\times 10^{-7}$. $E_{0}$ and $\upsilon$ are the pulse amplitude and central frequency ($E_{0}=1.5$ kV/cm).

**Figure 8:**
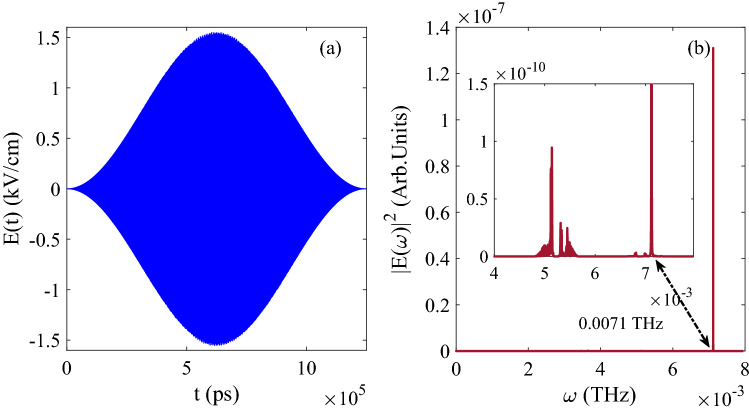
(**a**) The evolution of control pulse as function of time; (**b**) The Fourier transform of pulse in (**a**).

Then the optimized pulse is shown in Fig. 8a, and the Fourier transform of the pulse is shown in Fig. 8b, the amplitude of pulse is around $E_{0}=1.5 kV/cm$ and the central frequency is 0.0071 THz, which is the energy difference between 0.6337 cm$^{-1}$ and 0.3939 cm$^{-1}$ due to the quantum basis $\vert 01\rangle$ and $\vert 10\rangle$. Meanwhile, the pulse also satisfies the condition of resonance.

**Figure 9:**
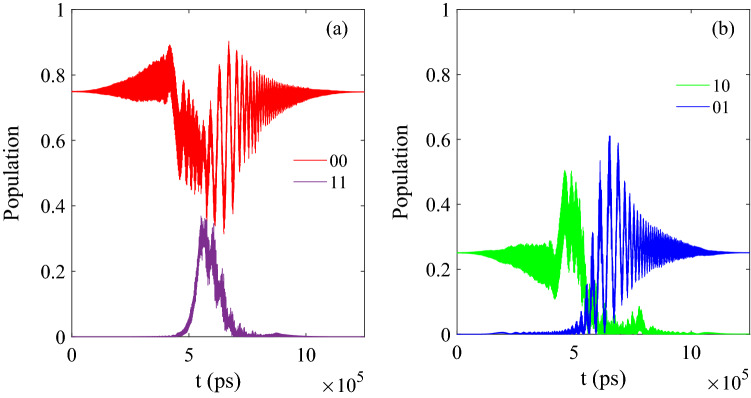
The evolution of population as function of time: (**a**) Corresponding to $\vert 00 \rangle$ and $\vert 11 \rangle$; (**b**) Corresponding to $\vert 10 \rangle$ and $\vert 01 \rangle$.

When the initial state is set as $[\sin (\pi /3)\vert 00 \rangle + \cos (\pi /3)\vert 10 \rangle ]e^{-i\phi }$, and the population of final state is 0.7487 for $\vert 00 \rangle$, 0.2505 for $\vert 01 \rangle$, $7.7637\times 10^{-4}$ for $\vert 10 \rangle$ and $4.6586\times 10^{-4}$ for $\vert 11 \rangle$, as the ideal value should be 0.75 for $\vert 00 \rangle$, 0.25 for $\vert 01 \rangle$, 0.00 for $\vert 10 \rangle$ and 0.00 for $\vert 11 \rangle$), and the evolution of population is shown in Fig. 9. From Fig. 9b, it can be known that the basis $\vert 10 \rangle$ and $\vert 01 \rangle$ exchange the population for each other, which satisfies the principle of the logical gate SWAP correctly. Meanwhile, the fidelity of the processing can also reach 0.999.

Based the same quantum basis and the molecular pendular system, we try to optimize the SWAP logical gate. Compared to the results in optimization of CNOT, it can be seen the energy difference of pendular system is more smaller than in latter, and it needs more time of evolution and intensity of pulse to achieve the ideal fidelity, and the more iterations and longer running time of Code are needed. In summary, both of two-qubit logical gates CNOT and SWAP can be generated ideally in this pendular system.

## Discussion

In conclusion, we applied two quantum optimal control methods to find optimized control fields for constructing two-qubit logical gates, specifically, CNOT and SWAP gates. In addition, the pendular states of trapped polar molecules SrO–SrO system are choosen as quantum basis which is analyzed in Ref.^28^. Both of the quantum gates we studied show good performance with high fidelities and ideal population were achieved and close to the theoretical values. The same specific initial states have been studied in this work, and the high fidelities 0.975 and 0.999 were achieved during the optimization for the above gates. Compared to the results in Ref.^28^, our results have demonstrated the higher fidelities and more perfect population which meant the more better optimization for quantum calculation. Meanwhile, the optimization methods is based on iterative solution for the coupled wave function equations, and the required convergence time and Random Access Memory (RAM) are also the important factors should be considered. According to the guidance of Ref.^28^, it was found that the convergence speed in MC-OCT is obviously faster than MT-OCT and the total evolution time have the same rule. Finally, the above two methods can be extended to optimized general quantum gates straightly. In practical experiment for specific systems, MC-OCT with constraints (e.g., pulse energy, fluence and magnitude) should be achieved much easier than MT-OCT.

It is also important to discuss analytical realization for experiment. Such as, in Ref.^51^ the authors propose and thoroughly investigate the scheme of employing trapped ultracold atoms in optical lattice to function as viable platform for quantum CN and CCN gates. In addition, they present a novel approximation method for realizing one- and two-qubit gates for the realization of quantum algorithms and their discussion can also be extended considering the dissipation effect in Ref.^52^. In Ref.^53^, the authors propose and analyze a detailed experimental procedure for implementing an N-bit discrete quantum Fourier transform. Furthermore, a new application of ballistic nanowires with spinbit interaction to realize some new quantum gates are discussed, such gates would be of interest to be experimentally implemented without scattering process in Ref.^54^. Based on the analysis, we hope the numerical simulation of our work can provide theoretical basis for experimental implementation.

## Methods

### Multi-target optical control theory

Much attention has recently been devoted to applying optimal control theory for elements of quantum computation in molecular systems. The basic idea is to design laser pulses which allow manipulation of transitions within each qubit separately. For implementing basic quantum gates, the aim is to achieve large transition probabilities with the correct phase from a specific initial state into a final target state by application of an external laser field while minimizing the laser energy. The objective function $\jmath$ in the optimal control theory for elements of quantum computation can be maximized^43,44^

12 $$\begin{aligned} \jmath \left[ \psi _{ik}(t),\psi _{fk}(t),E(t)\right]= & {} \sum _{k=1}^{z}\{\left| \left\langle \psi _{ik}(T)\right| \phi _{fk}\rangle \right| ^{2}-\alpha \int \nolimits _{0}^{T}\nonumber \frac{\left| E(t)\right| ^{2}}{ S(t)}dt \nonumber \\&-2{\mathrm {Re}}\{\left\langle \psi _{ik}(T)\right| \phi _{fk}\rangle \int \nolimits _{0}^{T}\left\langle \psi _{fk}(t)\right| \frac{i}{\hbar }[H-\mu \cdot E(t)]+\frac{\partial }{\partial t}\left| \psi _{ik}(t)\right\rangle \}. \end{aligned}$$

where $\psi _{ik}(t)$ and $\psi _{fk}(t)$ is the wave function driven by the optimal laser field, *E*(*t*). *z* is the total number of targets and $z = 2N + 1$ is set for *N* qubits. 2*N* is the number of input-output transitions in the gate transformation, and the supplementary equation is the phase constraint. A small $\triangle \varphi$ can be got by laser pulses during optimization produces and has a weak dependence on the initial qubit state^7,55^. $\alpha$ is the Lagrange multiplier, and the envelope function *S*(*t*) is $\sin ^{2}(\pi t/T)$ $\sin ^{2}(\pi t/T)$. *H* is the total Hamiltonian of system and $H=H_{0}-\mu \epsilon (t)$, $H_{0}$ is Hamiltonian of rotational system and $\epsilon (t)$ which makes the rotational state transfer to pendular states composed of two polar molecules. $\mu$ is the transition dipole moment and *T* is the total evolution of time.

In order to get the optimized control field, $\delta \jmath =0$ should be satisfied. And the coupled Schrödinger equations with the control of laser pulse are written as

13 $$\begin{aligned} i\hbar \frac{\partial }{\partial t}\psi _{ik}(t)= & {} \{H-\mu \cdot E(t)\}\psi _{ik}(t), \nonumber \\ i\hbar \frac{\partial }{\partial t}\psi _{fk}(t)= & {} \{H-\mu \cdot E(t)\}\psi _{fk}(t), \nonumber \\ \psi _{ik}(0)= & {} \phi _{ik}, \nonumber \\ \psi _{fk}(T)= & {} \phi _{fk}, \nonumber \\ k= & {} 1...z. \end{aligned}$$

where $\phi _{ik}$ is the wave function of $\psi _{ik}$ at the initial time and $\phi _{fk}$ is the wave function of $\psi _{fk}$ at the final time. *k* is the number of equations. The control pulse has the specific form

14 $$\begin{aligned} E(t)= & {} -\frac{z\cdot \mu \cdot S(t)}{\hbar \cdot \alpha _{0}}\cdot \sum \limits _{k=1}^{z}{\mathrm {Im}}\mathbf {\{}\left\langle \psi _{ik}(t)\right| \psi _{fk}(t)\rangle \nonumber \\&\left\langle \psi _{fk}(t)\right| \cos \theta _{1}+\cos \theta _{2}\left| \psi _{ik}(t)\right\rangle \mathbf {\}}. \end{aligned}$$

where $\theta _{1}$ and $\theta _{2}$ is the angular between the axiex of two molecules and the static external electric field $\epsilon (t)$. By solving partial differential equations, the evolution time varies from 0 to final T. During this processing, the control pulse are optimized and the population and fidelity reach the ideal accuracy. Here, the population of wave function is written as

15 $$\begin{aligned} P=\frac{1}{z}\cdot \sum _{k=1}^{z}\left| \left\langle \psi _{ik}(T)\right| \psi _{fk}(t)\rangle \right| ^{2}, \end{aligned}$$

the fidelity is defined as

16 $$\begin{aligned} F=\frac{1}{z^{2}}\cdot \left| \sum _{k=1}^{z}\left\langle \psi _{ik}(T)\right| \psi _{fk}(t)\rangle \right| ^{2}. \end{aligned}$$

Based on the above the simulation, the first split method and the second split method are used to solve the time-dependent Schrödinger equation, the latter is more precisely^43,44^.

### Multi-constraint optical control theory

The time evolution of wave function for the initial state $|i\rangle$ can be described by $\Psi (t)=\hat{U}(t,0)\vert i \rangle$ with a unitary evolution operator $\hat{U}(t,0)$, which is governed by time-dependent Schrödinger equation^46,48^

17 $$\begin{aligned} i\dfrac{\partial \hat{U}(t,0)}{\partial t}=\hat{H}(t)\hat{U}(t,0), \hat{U}(0,0)={\mathbb {I}}, \end{aligned}$$

with $\hslash =1$ (atomic units are used in this work). The goal of this work is to optimize control field *E*(*t*) to generate a specified unitary transformation and implement desired logic gate based on specific molecular system, so that a specified unitary transformation *H* can be realized with the final unitary operator $\hat{U}_{T}=\hat{U}(T,0)$. A convenient mathematical formulation of this control objective (fidelity) is

18 $$\begin{aligned} F(U_{T})=\dfrac{1}{2^{n}} \vert {\mathrm{Tr}}(W^{\dagger }U_{T}) \vert ^{2}= \dfrac{1}{2^{n}} \langle W \vert U_{T}\rangle \langle U_{T}\vert W \rangle , \end{aligned}$$

where $\langle W \vert U_{T} \rangle ={\mathrm{Tr}}(W^{\dagger }U_{T})$, $F(U_{T}) \in [0,1]$ and *n* is the number of qubits, then $n=2$ means the two-qubit gate (CNOT) in this work.

To optimize the control field for maximizing the fidelity, a dummy variable $s\geqslant 0$ as used in Ref.^48^ is employed to parameterize the optimization, which can be expressed as

19 $$\begin{aligned} g_{0}(s)\equiv \dfrac{dF(s,U_{T})}{ds}=\int ^{T}_{0} \dfrac{\delta F(s,U_{T})}{\delta E(s,t)} \dfrac{\partial E(s,t)}{ \partial s} dt\geqslant 0, \end{aligned}$$

that can be satisfied by updating the control pulse as

20 $$\begin{aligned} \dfrac{\partial E(s,t)}{ \partial s} = \dfrac{\delta F(s,U_{T})}{\delta E(s,t)}. \end{aligned}$$

In the resonant optical control case^46,47^, the interaction between the dipole-dipole molecule and control field plays an important role, the fidelity can be written as

21 $$\begin{aligned} \dfrac{\delta F}{\delta E(t)}= & {} \dfrac{1}{2^{n}} \left( \langle H \vert \dfrac{\delta U_{T}}{\delta E }\rangle \langle U_{T} \vert H \rangle + \langle H \vert U_{T} \rangle \langle \dfrac{\delta U_{T}}{\delta E }\vert H \rangle \right) \nonumber \\= & {} \dfrac{1}{2^{n}} \left( \langle H \vert -i\mu (t) \rangle \langle U_{T}\vert H \rangle + \langle H \vert U_{T} \rangle \langle -i\mu (t) \vert H \rangle \right) \nonumber \\= & {} \dfrac{1}{2^{n}} 2 \Re \left( \langle H \vert -i\mu (t)\rangle \langle U_{T}\vert H \rangle \right) . \end{aligned}$$

where $\hat{\mu }(t)=\hat{U}^{+}(t,0) \mu \hat{U}(t,0)$, *F* represents the fidelity in resonance.

For practical applications, Eq. (20) may be generalized to include a set of equality constraints $F(E(\cdot ,s))$, on the optimal optical fields

22 $$\begin{aligned} g_{\ell }(s)\equiv \frac{dF (E(\cdot ,s))}{ds}=\int _{0}^{T} \frac{\delta F(E(\cdot ,s))}{\delta E(s,t)}\frac{\partial E(s,t)}{\partial s}dt=0. \end{aligned}$$

The combined demands in Eqs. (19) and (22) can be fulfilled simultaneously by updating the control field as function of variable *s*

23 $$\begin{aligned} \dfrac{\partial E(s,t)}{ \partial s} =S(t)\sum _{\ell =0}^{M}[\Gamma ^{-1}]_{0\ell }\frac{\delta F}{\delta E(t)}, \end{aligned}$$

where $S(t)\ge 0$ is an envelope function which smoothly turns on and off the control field, and $\Gamma$ is an invertible full rank $(M+1)\times (M+1)$ square matrix composed of elements

24 $$\begin{aligned} \Gamma _{\ell +1,\ell '+1}=\int _{0}^{T}S(t)\frac{\delta F}{\delta E(t)}\frac{\delta F'}{\delta E(t)}dt. \end{aligned}$$

Here the optimized control field is limited to satisfy two constraints simultaneously

25 $$\begin{aligned} c_1\equiv \int ^{T}_{0} E(s,t)dt = 0, \end{aligned}$$

and

26 $$\begin{aligned} c_2\equiv \int ^{T}_{0} E^{2}(s,t)dt = {\mathcal {C}}. \end{aligned}$$

The zero-pulse area constraint in Eq. (25) implies that the optimized control field does not contain dc-components, leading to pure ac control, whereas the constant fluence constraint in Eq. (26) keeps the energy of the optimized fields unchanged as compared with the initial guess. The numerical details of performing this multiple constraint quantum optimal control method can be found in previous works^46,47^.

## Data Availability

The datasets used and/or analysed during the current study available from the corresponding author on reasonable request.
